# Supplementary figures and images for: Cells Engage Endogenous Malonate Synthesis to Drive Mitochondrial Metabolism
Source: bioRxiv. 2026 May 29:2026.05.22.727248. Preprint. [Version 2] doi: 10.64898/2026.05.22.727248 (PMC13228397; doi:10.64898/2026.05.22.727248)

## Figure S1

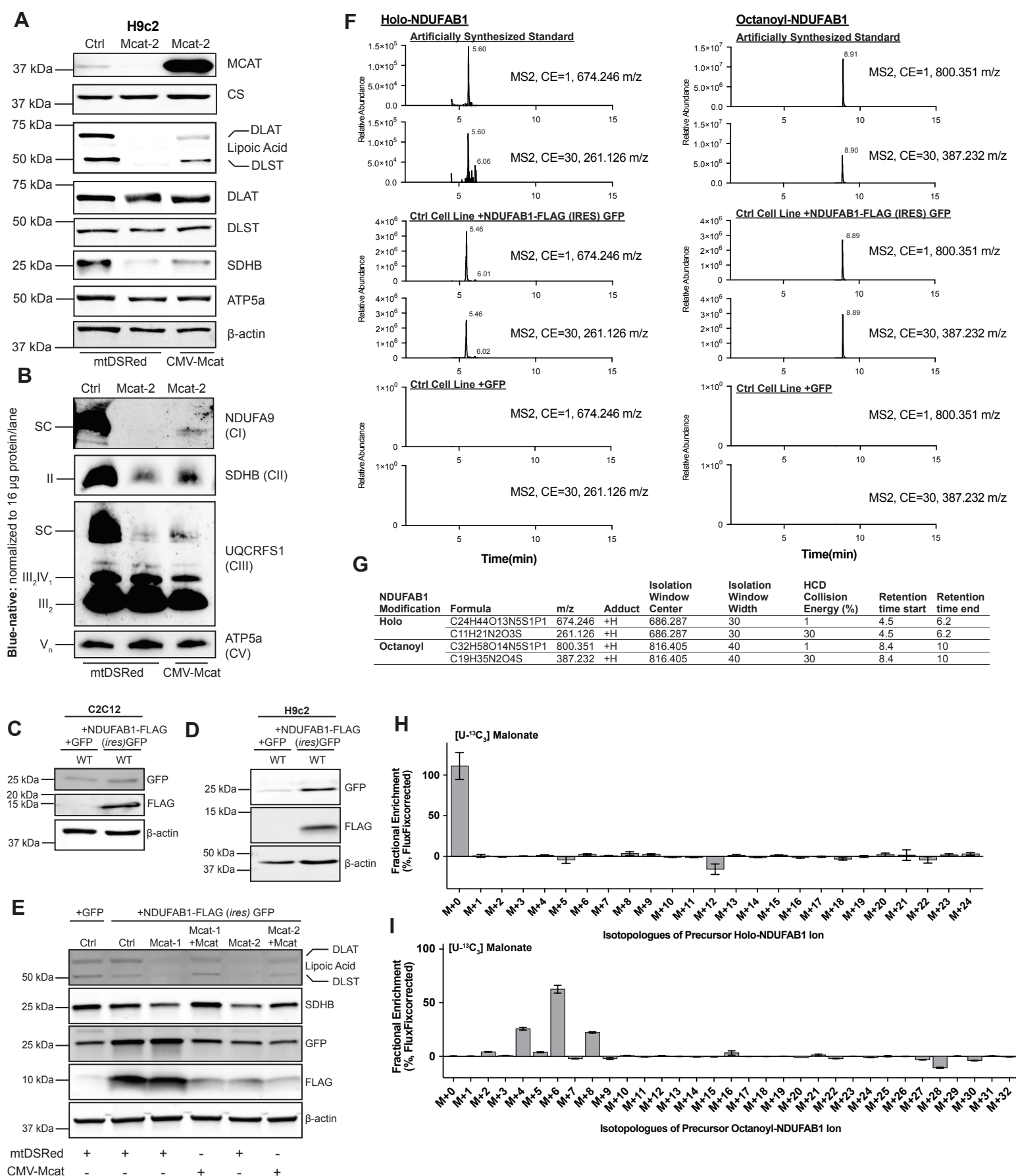

## Figure S2

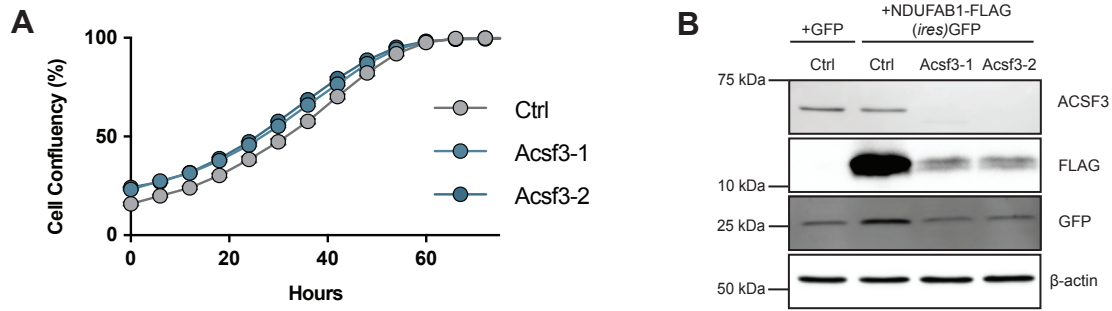

**Figure S3**

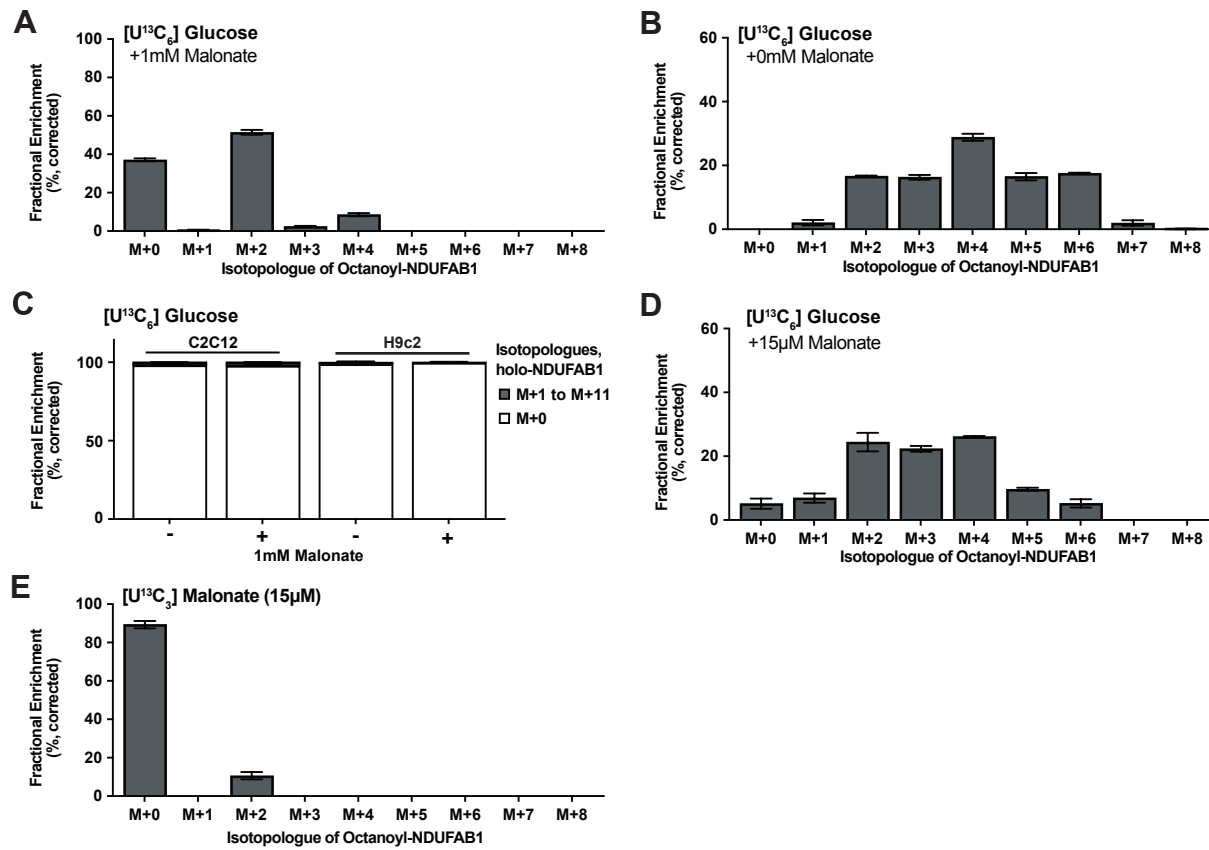

**Figure S4**

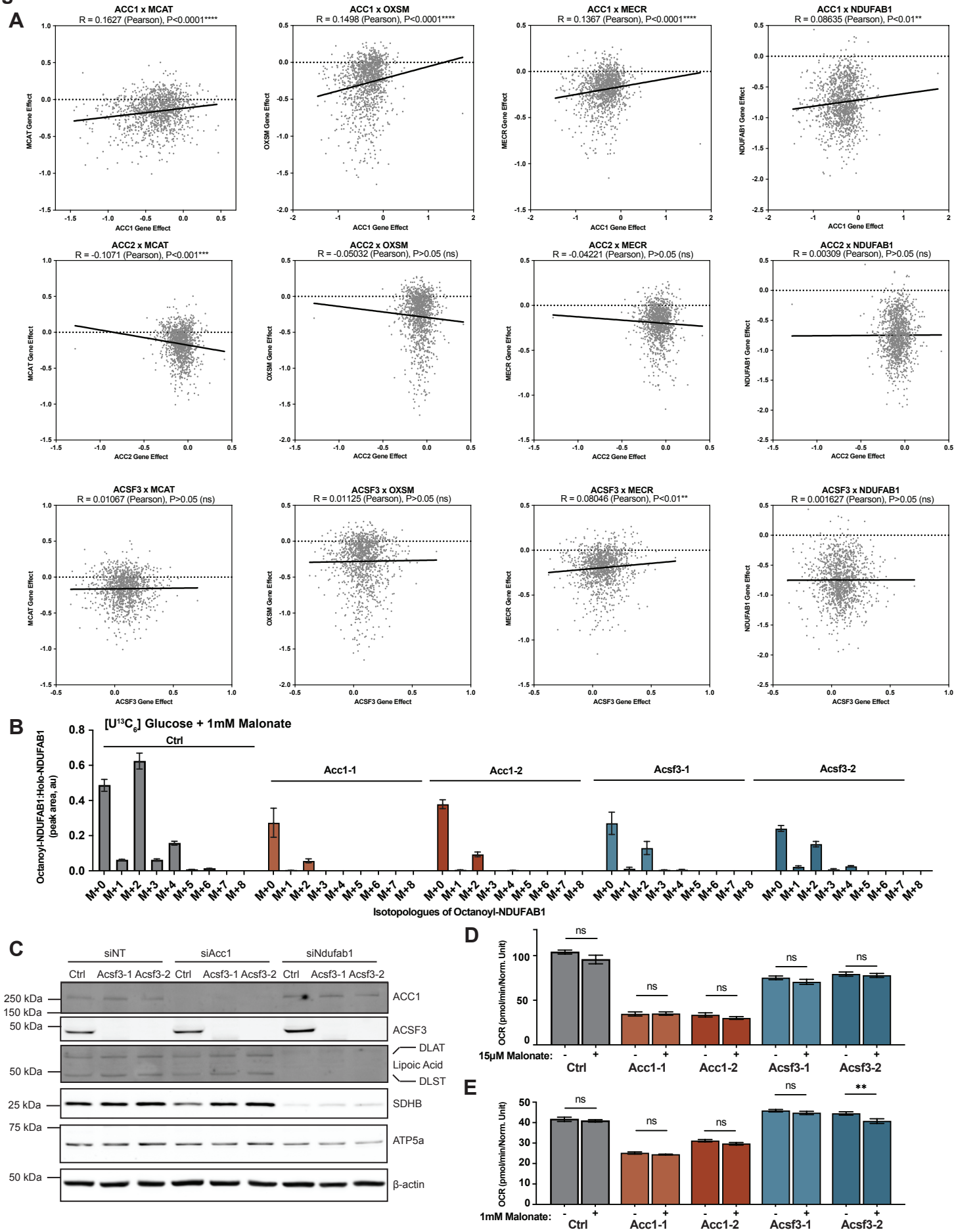

Supplement: Supplement 1 — Figure S1. Malonate Drives Mitochondrial Fatty Acid Synthesis. (A) Whole cell lysates or mitochondrial enriched lysates (MCAT, CS) in clonal H9c2 cell lines of the indicated genotypes. (B) Blue-native PAGE of crude isolated mitochondrial lysates from the indicated H9c2 cell lines expressing mtDSRed or Mcat, immunoblotted for the indicated ETC complex subunits. Data are representative of 3 biological replicates. (C-E) Whole cell lysates from (C) wild-type C2C12 cells or (D) wild-type H9c2 cells or (E) C2C12 cells of the indicated genotype expressing mtDSRed or Mcat, expressing GFP or NDUFAB1-FLAG(ires)GFP blotted with the indicated antibodies. (F) Extracted ion chromatograms of ions corresponding to holo-NDUFAB1 or octanoyl-NDUFAB1 from the indicated sample and HCD collision energy (%). (G) Table of quantified ions for the indicated NDUFAB1 modification after AspN digest and the corresponding approach by mass spectrometry for detection in a 15-minute C18 reversed phase liquid chromatography method. (H) Mass isotopologue distribution of precursor ion for holo-NDUFAB1 detected in control cell line (n=3) after culture in uniformly labeled 13C malonate for 3 hours. (I) Mass isotopologue distribution of precursor ion for octanoyl-NDUFAB1 detected in a control cell line (n=3) after culture in uniformly labeled 13C malonate for 3 hours, corrected with unlabeled sample. Error bars represent +/− 1 SEM. Figure S2. Malonate Drives Mitochondrial Fatty Acid Synthesis (A) Cells of the indicated genotype seeded in 6-well plates and incubated for the indicated time and assessed for confluency by Incucyte image analysis (n=3). Representative of three biological replicates. (B) Whole cell lysates separated by SDS-PAGE of the indicated cell line, immunoblotted with the indicated antibodies. Representative of three biological replicates. Error bars represent +/− 1 SEM. Figure S3. Under physiologic conditions cells engage malonate synthesis to support mtFAS. (A) Mass isotopologue distrib [file NIHPP2026.05.22.727248v2-supplement-1.pdf]
